# Contrast-Enhanced Mammography (CEM) for Detecting Residual Disease after Neoadjuvant Chemotherapy: A Comparison with Breast Magnetic Resonance Imaging (MRI)

**DOI:** 10.1155/2018/8531916

**Published:** 2018-11-08

**Authors:** Filipe Ramos Barra, Alaor Barra Sobrinho, Renato Ramos Barra, Mayra Teixeira Magalhães, Laira Rodrigues Aguiar, Gabriela Feitosa Lins de Albuquerque, Rodrigo Pepe Costa, Luciano Farage, Riccardo Pratesi

**Affiliations:** ^1^Department of Radiology and Molecular Imaging, Imagens Médicas de Brasília, Brasília, DF, Brazil; ^2^Breast Service, Department of Surgery, Hospital de Base do Distrito Federal, Brasília, DF, Brazil; ^3^Graduate Program in Health Sciences, School of Health Sciences, University of Brasília, Brasília, Brazil; ^4^School of Medicine, University of Brasilia, Brasília, DF, Brazil

## Abstract

**Objective:**

To evaluate the performance of contrast-enhanced mammography (CEM) compared to magnetic resonance imaging (MRI) for estimating residual tumor size after neoadjuvant chemotherapy (NAC) in women with newly diagnosed breast cancer.

**Methods:**

The institutional review board approved this study. This prospective study included women with newly diagnosed breast cancer who underwent breast CEM and MRI at the end of the last cycle of NAC and before definitive surgery. Size of residual malignancy on post-NAC CEM and MRI was compared with surgical pathology. Agreements and correlations of CEM and MRI measurements with histological size were assessed.

**Results:**

Thirty-three patients were included with a mean age of 45 years (range 22–76). The sensitivity, specificity, and positive and negative predictive value for detection of residual disease of CEM were 76%, 87.5%, 95%, and 86.4%, and those of MRI were 92%, 75%, 92%, and 75%. Comparing CEM to MRI, the mean difference was −0.8 cm, concordance coefficient was 0.7, and Pearson correlation was 0.7 (p = 0.0003). The concordance coefficient between measurements of each imaging modality and pathologic tumor size was 0.7 for CEM and 0.4 for MRI. Pearson correlation was 0.8 for CEM and 0.5 for MRI. Mean differences between CEM, MRI, and residual histopathological tumor size were 0.8 cm and 1.8 cm, respectively.

**Conclusions:**

CEM has good correlation and agreement with histopathology for measuring residual disease after NAC. CEM was comparable to MRI, showing high positive predictive value and specificity for detecting residual disease.

## 1. Introduction

Neoadjuvant chemotherapy (NAC) was used as a primary therapeutic strategy for locally advanced and inflammatory breast cancers. It may decrease the extent of the tumor, increasing the chances of successful breast-conservation surgery, and provide prognostic information to evaluate treatment response [1].

Response to NAC is currently assessed by combining clinical examination and conventional imaging techniques such as mammography, breast magnetic resonance (MRI), and ultrasound (US). To date, breast MRI has proven to be superior to mammography and US in the assessment of tumor extent and presence of additional foci and highly sensitive in identifying residual disease following NAC [2–6]. Unfortunately, the limited availability of MRI and its high cost may restrict its access for patients.

Contrast-enhanced mammography (CEM) is a new imaging tool that uses a dual-energy technique to combine the benefits of digital mammography with intravenous contrast utilization. In the diagnostic setting, CEM has a higher sensitivity for breast cancer detection compared with the sensitivities of full-field digital mammography (FFDM) alone and FFDM combined with ultrasound [7–16]. For assessing tumor extent, CEM findings were reported to have good correlation with histopathology size, even in dense breasts, and this correlation was better than those for FFDM and US [17]. CEM has been shown to detect breast cancer with a diagnostic accuracy that is comparable or even better than breast MRI, even in dense breasts [7, 8, 15, 16].

We aimed to evaluate the accuracy of CEM in assessing residual disease extent and predicting pathologic complete response (pCR) compared to MRI after completion of NAC in patients with a diagnosis of breast cancer.

## 2. Subjects and Methods

### 2.1. Study Design and Population

This prospective study was conducted to evaluate the performance of CEM for the diagnosis of residual breast cancer after NAC. This study enrolled patients who underwent NAC followed by surgery with curative intent for locally advanced primary breast cancer between August 2015 and December 2017. The eligibility criterion was pathologically confirmed breast cancer by core biopsy. Patients were excluded if they had (a) a pacemaker, (b) previous allergic reaction to contrast media, or (c) history of breast cancer treated with chemotherapy. The study was approved by the local ethics committee, and informed consent was obtained from all patients.

### 2.2. Imaging Technique

#### 2.2.1. CEM

Bilateral craniocaudal (CC) and mediolateral oblique (MLO) views were acquired using a commercially available FFDM system (GE Senographe DS, GE Healthcare, Milwaukee, WI). A pair of low- and high-energy images during single-breast compression was obtained per view. Before image acquisition, 1.5 ml/kg of non-ionic contrast medium (Iohexol, 300 mg/ml) was injected using an automated injector at a flow rate of 3 ml/s. The first image was obtained 1.5–2 min after injection initiation, and all images were obtained within 4–7 min. The complete examination protocol has been previously described and explained [18].

#### 2.2.2. Breast MRI

All breast MRI examinations were performed using a 1.5T MRI system (GE Signa HDxT, GE Healthcare) with a dedicated eight-channel breast coil. The following sequences were acquired: axial 2D T1W fast spoiled gradient echo (flip 90°, TR/TE 467.0/11.0 ms, bandwidth 62.5 kHz, FOV 20 cm × 20 cm, matrix 384 × 384, slice/gap 4/0.4 mm); axial 2D Short-TI Inversion Recovery (flip 90°, TR/TE/TI 6000/32/150 ms, bandwidth 31.25 kHz, FOV 36 cm × 36 cm, matrix 320 × 192, slice/gap 5/1 mm); axial 2D diffusion weighted imaging SE EPI (b value = 0 and 750 s/mm^2^, TR/TE 12500/78.2 ms, bandwidth 250 kHz, FOV 32 cm × 32 cm, matrix 256 × 192, slice/gap 4/0.4 mm); sagittal 3D T1W fat suppressed volume imaging for breast assessment (VIBRANT) (flip 15°, TR/TE/TI 4.8/1.8/7.0 ms, bandwidth 50 kHz, FOV 24 cm × 20 cm, matrix 352 × 256, slice/gap 3/0 mm) acquired dynamically (1 pre- and 3 post-gadolinium injection phases) with a 87 s temporal resolution; and high spatial resolution post-contrast T1W fat suppressed 3D VIBRANT (flip 15°, TR/TE/TI 4.6/2.1/16.0 ms, bandwidth 62.5 kHz, FOV 32 cm × 32 cm, matrix 320×320, slice/gap 1/0mm). Contrast medium (0.1 mmol/kg of gadodiamide) was injected with a 10-second timing delay into the antecubital vein using an 18 to 20-gauge needle at a flow rate of 2 mL/s followed by a flush of 20 ml of saline solution. The average acquisition time was 30 min.

### 2.3. Imaging Interpretation

Images were interpreted independently by a trained radiologist with more than five years of experience with both CEM and FFDM and breast MRI interpretation. A dedicated workstation (Advantage Workstation 4.7, GE Healthcare) was used for interpretation of MRI studies. CEM examinations were interpreted on 5-megapixel monitors at full resolution with one breast on each monitor using a dedicated mammography software program (Seno Iris, GE Healthcare).

The response evaluation criteria in solid tumors (RECIST), version 1.1 [19] was used to assess the tumor response to treatment. Response was classified as follows: complete response (CR, disappearance of all lesions), partial response (PR, ≥ 30% dimensional reduction), stable disease (SD, < 30% dimensional reduction or < 20% dimensional increase), and progressive disease (PD, ≥ 20% dimensional increase). In CEM, measurements were obtained on recombined images, based on contrast uptake and, in MG, on low-energy images. In MRI, the maximum diameter of the enhancing lesion was measured on first post-contrast sagittal VIBRANT images. A radiologist visually assessed the mammographic density of the breasts on low-energy images.

### 2.4. Histopathological Evaluation

All patients underwent breast-conserving surgery or mastectomy after completion of NAC, and surgical specimens were evaluated by a pathologist for the size of residual malignancy. Surgical pathology reports were reviewed for the following parameters: histologic tumor subtype, tumor size, estrogen receptor, progesterone receptor, and HER2 statuses. Tumor size was defined as the largest dimension based on macroscopic and histopathological examination. In the case of multifocal breast cancer, a sum of the diameters for all target lesions was calculated. We considered pCR as the absence of invasive cancer in the breast irrespective of ductal carcinoma in situ or nodal involvement (ypT0/is).

### 2.5. Statistical Analysis

Tumor sizes measured by CEM, FFDM, and MRI were compared to the tumor size measured in pathology (reference standard). Also, CEM and FFDM results were compared to MRI results. Agreements of FFDM, CEM, and MRI measurements with histological size were assessed using Bland–Altman plots. Lin's concordance and Pearson correlation coefficient were used to assess agreement between diagnostic imaging tools and pathology results. The sensitivity of a test was defined as the proportion of patients with a positive study among those with residual tumors. Specificity was defined as the proportion of patients with a negative study among those without residual tumors. Statistical analyses were performed using MedCalc for Windows, version 14.8.1 (MedCalc Software, Belgium). A p value <0.05 was considered significant.

## 3. Results

### 3.1. Patient and Tumor Characteristics

A total of 33 women were included with a mean age of 45 years (range, 22–76 years). Regarding menstrual status, 27 (81.8%) of the women were premenopausal and 6 (18.2%) were postmenopausal. A total of 22 (66.7%) had heterogeneously dense breasts or dense breasts. The majority underwent mastectomy (n=24; 72.7%). The mean time intervals between studies and surgery were 40 days for CEM and FFDM and 47 days for MRI. The mean time interval between the end of NAC and surgery was 55 days (range, 20-122 days).

Histological results comprised invasive ductal carcinoma (n=29; 87.9%), mixed invasive carcinoma (n=3; 9.1%), and invasive lobular carcinoma (n=1; 3%). The molecular subtype distribution in descending order was as follows: luminal B (n=16; 48.5%), luminal A (n=6; 18.2%), triple negative (n=10; 30.3%), and HER2-Overexpressing (n=1; 3%). Detailed information of each women included in the study is described in Table 1.

### 3.2. Tumor Size Assessment

Pathologic mean tumor size after NAC was 1.6 cm (range 0–7.5 cm) compared with 2.4 cm (range 0–13.1 cm) for CEM, 2.4 cm (range 0-7.2 cm) for FFDM, and 3.6 cm (range 0–12.5 cm) for MRI. CEM accurately showed final tumor size to within 1 cm in 23 patients (69.7%) and overestimated tumor size by more than 1 cm in 8 patients (24.2%). FFDM accurately showed final tumor size to within 1 cm in 13 patients (39.4%) and overestimated tumor size by more than 1 cm in 15 patients (45.4%). MRI accurately showed final tumor size to within 1 cm in 12 patients (36.3%) and overestimated tumor size by more than 1 cm in 19 patients (57.6%).

**Figure 1:**
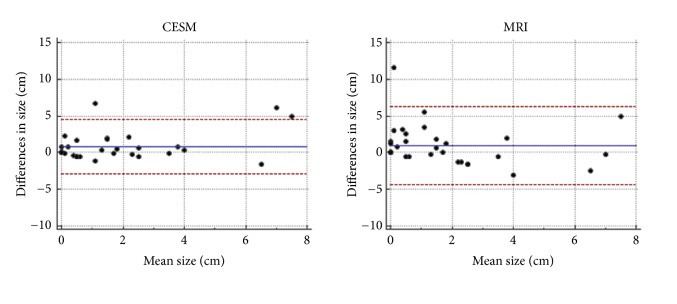
Bland-Altman plots comparing measurements of residual tumor with CEM (left) and MRI (right) compared to surgical specimen.

The concordance coefficient between measurements of each imaging modality and pathologic tumor size was 0.7 for CEM, 0.3 for FFDM, and 0.4 for MRI. Pearson correlation was 0.8 for CEM, 0.3 for FFDM, and 0.5 for MRI. Mean differences between CEM, FFDM, MRI, and residual histopathological tumor size were 0.8 cm, 0.7 cm, and 1.8 cm, respectively. Limits of agreement and more detailed information are described in Table 2 and Figure 1.

**Figure 2:**
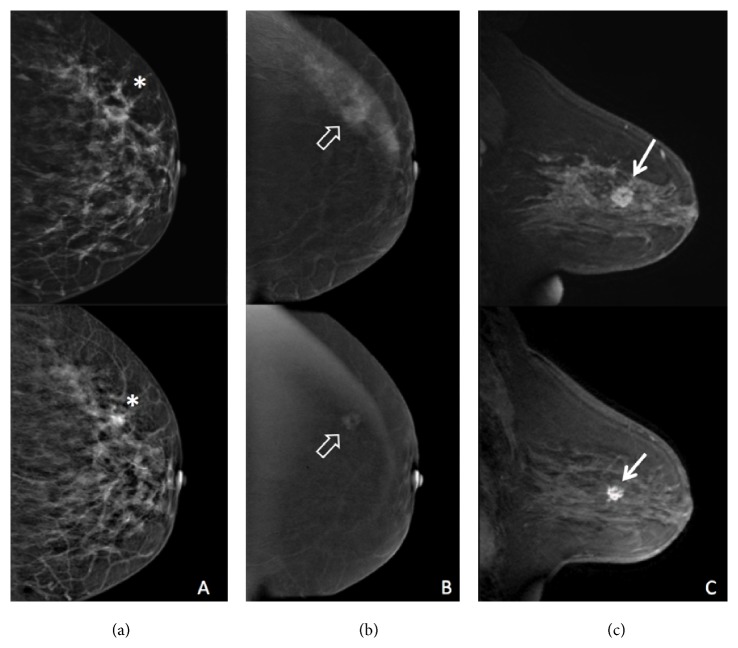
42-year-old woman. Invasive ductal carcinoma after neoadjuvant chemotherapy with residual disease. Pre-NAC (top row) and post-NAC (bottom row) FFDM (a), CEM (b), and MRI (c). A mass with distortion calcifications can be seen on FFDM (*∗*). Mass enhancement with irregular margins is shown on CEM (open arrow) and MRI (arrow).

**Figure 3:**
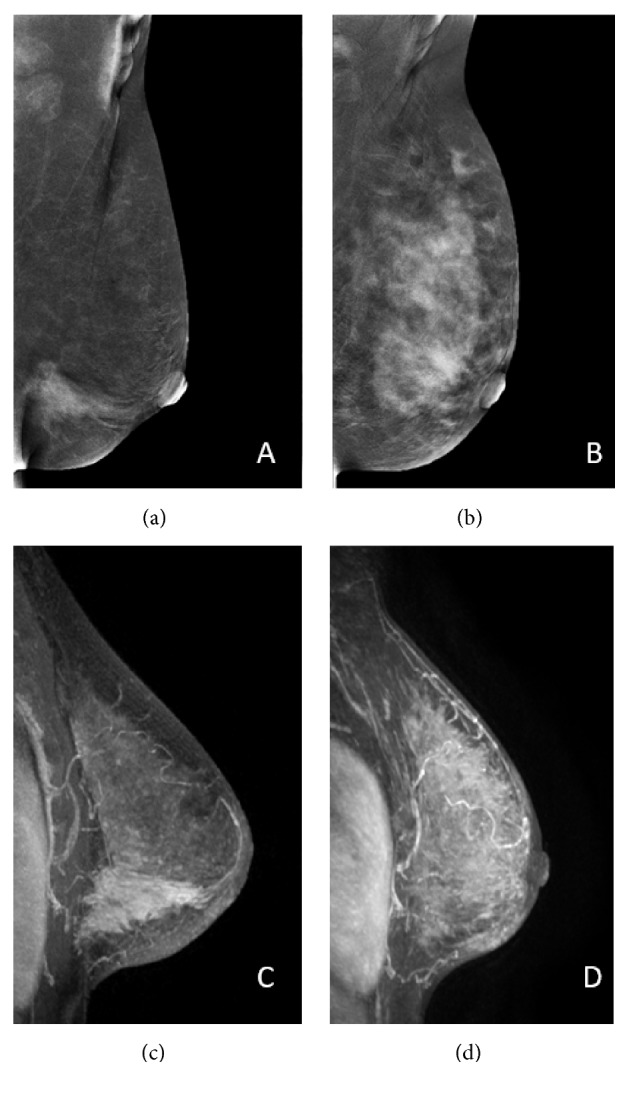
34-year-old woman. Invasive ductal carcinoma with progressive disease. Segmental non-mass enhancement is detected in the lower inner quadrant of the left breast on contrast-enhanced mammography (a) and magnetic resonance imaging (c) before NAC. After NAC, a diffuse non-mass enhancement is detected on CEM (b) and MRI (d).

Among the 25 patients who had RD, 19 were positive by CEM and FFDM, and 23 were positive by MRI. Progressive disease on imaging was correctly described on CEM and MRI (Figure 2), in two women, one of them with false negative post-NAC FFDM (Figure 3). Higher sensitivity was found by MRI, 92%, followed by CEM and FFDM, both 76% (Table 3). Positive predictive value (PPV) for the detection of residual disease was 95% for CEM, 86.4% for FFDM, and 92% for MRI. Negative predictive value (NPV) was 53.8% for CEM, 45.4% for FFDM, and 75% for MRI.

**Figure 4:**
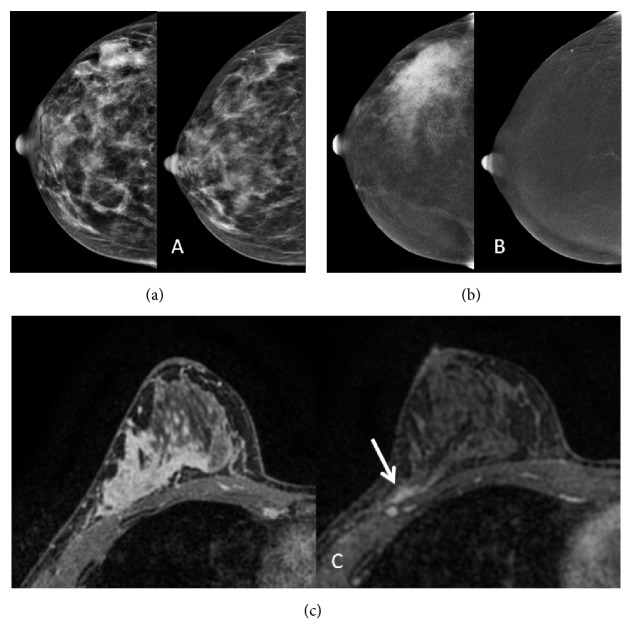
39-year-old woman. Invasive ductal carcinoma with pathologic complete response. Pre- and post-NAC FFDM (a), CEM (b), and MRI (c). Focal asymmetry is seen in the outer quadrant in FFDM (a). Pre-NAC CEM (b) and MRI (c) show segmental non-mass enhancement. Post-NAC FFDM (a) and MRI (c) incorrectly showed residual disease (arrow) while CEM was truly negative.

**Figure 5:**
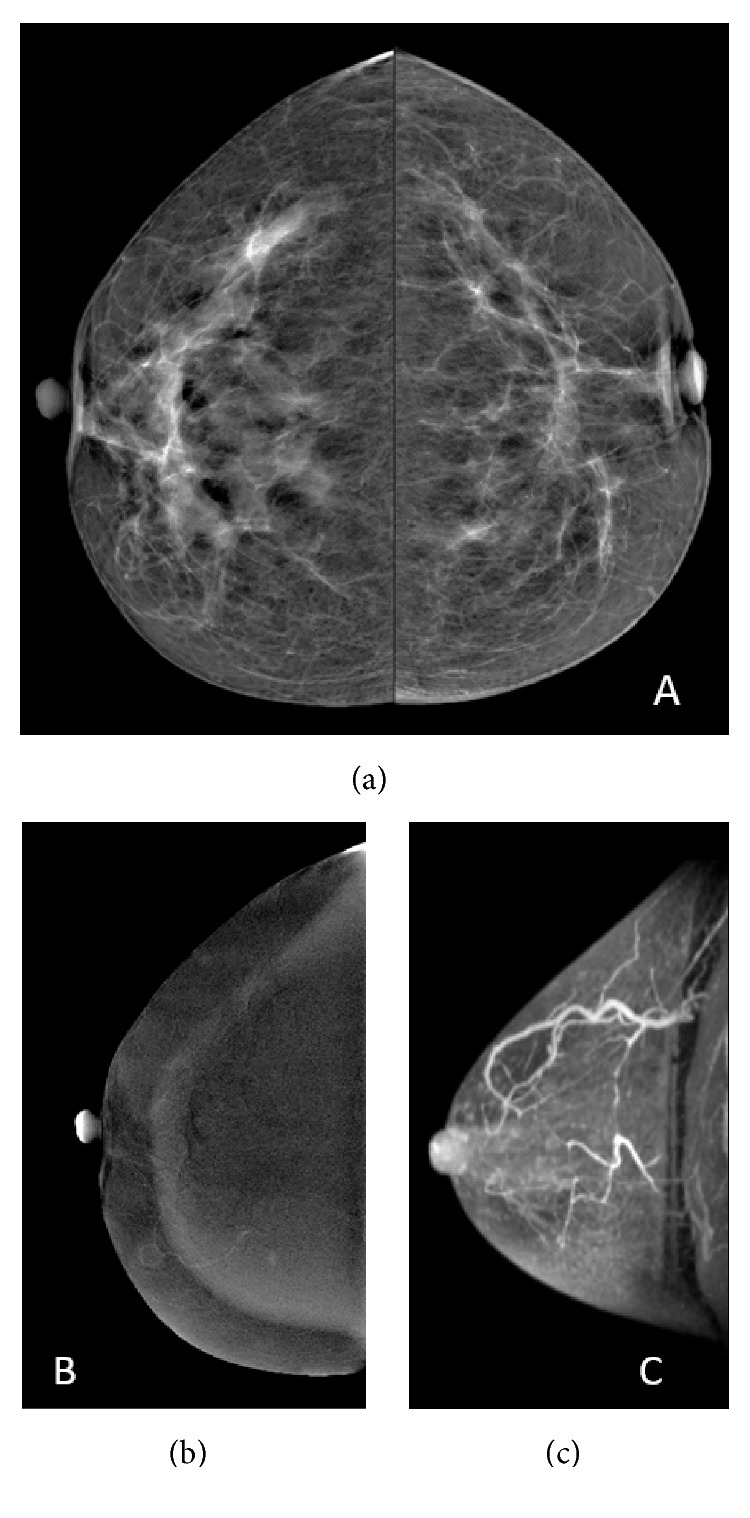
52-year-old woman. Invasive ductal carcinoma with pathologic complete response. Focal asymmetry is detected in the upper outer quadrant of the right breast (a). Contrast-enhanced mammography (b) and magnetic resonance imaging (c) without detectable lesions.

Pathologic complete response was achieved in 8 cases (24.2%); all of those were IDC, 6 (62.5%) luminal B, and 3 (37.5%) triple negative. Radiologic complete remission rate was 13 women in CEM (39.4%), 11 in FFDM (33.3%), and 6 in MRI (18.2%). Among the eight patients who had pCR, only one was positive by CEM for residual disease, three were positive by FFDM, and two were positive by MRI (Figure 4). The specificity of CEM to identify pCR was 87.5%, better than what was found by MRI, 75% (Table 3). On FFDM, three focal asymmetries were incorrectly assessed as residual disease (1.2 cm and 1.7 cm) (Figure 5).

### 3.3. Comparison between CEM and MRI

Mean difference between CEM and MRI was -0.8 cm (SD = 2.8). Lin's concordance coefficient was 0.7 (95% CI 0.4–0.8), and Pearson correlation was 0.7 (p = 0.0003). Limits of agreement were -6.3 to 4.7 cm between modalities. In comparison to MRI, CEM had measurements within 1 cm in 54.5% and overestimated size by more than 1 cm in 31.8% of the cases. All patients reported as having a complete radiologic response by MRI had negative CEM studies.

## 4. Discussion

The accurate assessment of residual disease at the end of neoadjuvant chemotherapy is imperative for surgical planning. Currently, MRI is accepted as the best imaging method for treatment monitoring [2, 3, 6]. CEM has been proposed as an alternative to MRI due to its shorter exam time, better tolerance, lower price, and reading time. CEM has been demonstrated to be comparable to MRI regarding cancer detection and evaluation of disease extent [8, 13–16].

In our study, we evaluated CEM for detecting and measuring residual tumor after NAC. All imaging methods overestimated residual tumor size in most patients. Difference between CEM and histopathological results was within 1 cm in almost 70% of the cases. A similar difference was reported in a recent study with patients submitted to neoadjuvant chemotherapy and endocrine therapy [20]. Limit of agreement with the residual tumor was narrower for CEM and wider for MRI, the opposite of what was found by Patel and colleagues [20]. It should be explained because in our study MRI overestimated residual tumor size in almost 60% of the cases. CEM had a good concordance coefficient and a good correlation with pathologic tumor size. Similar Lin's coefficient close to 0.8 was reported in studies performed in the United States and Italy [20, 21].

MRI demonstrated the best sensitivity and area under the receiver operator characteristic curve (AUC) for detecting residual tumor after NAC. CEM showed better specificity and PPV than MRI and FFDM. As well as our results, high specificity and PPV of CEM were also reported in women with locally advanced breast cancer [20, 22]. Despite demonstrating a higher specificity, Patel and colleagues found similar PPV in a group of older women when compared to the population of our study. A positive CEM, performed at the end of NAC, is highly associated with residual tumor at pathology and could help in the treatment decision.

Our results showed that when MRI was used as the reference, CEM showed good correlation and concordance (Figures 2, 3, and 5). All women with no residual enhancement on MRI had negative CEM studies. Published studies also found concordance and correlation among CEM and MRI post-NAC [20, 21].

This study had potential limitations. It was a single institution trial, and the small study population and uncommon molecular subtype distribution may limit the applicability of our results to the general population. For better comparison to MRI, CEM analysis was based only on recombined images. There is the risk of losing calcifications without enhancement, which may underestimate residual tumor measurements. Nevertheless, in our study, it was not identified. Although the radiologist was blinded to the results of other imaging modality when reviewing CEM and MRI, all studies were reviewed by the same radiologist, thereby introducing inherent bias. We were not able to evaluate the relationship of NAC agents, tumor histology, and molecular subtype with the performance of CEM.

## 5. Conclusions

CEM has good correlation and agreement with histopathology for measuring residual tumors after NAC. CEM is as reliable as MRI in assessing the presence of residual tumor. Our findings are encouraging since CEM might be useful for patients with contraindications to MRI and patients in regions with limited MRI availability or lack of reimbursement.

## Figures and Tables

**Table 1:** Characteristics of the studied population.

**Breast Density**	**Histological type**	**Molecular type**	**NAC Regimen**	**CEM Response**	**FFDM Response**	**MRI Response**	**Histopathological response**
ACR c	IDC	Luminal B	TAC	PR	PR	PR	pPR
ACR d	IDC	Luminal B	AC-T	CR	PR	PR	pCR
ACR d	IDC	Luminal B	AC-T	CR	CR	CR	pPR
ACR c	Mixed	Luminal B	FAC	PR	PR	PR	pPR
ACR d	IDC	Luminal A	AC-T	SD	SD	SD	pSD
ACR b	IDC	Her2+	TCH	PR	SD	PR	pPR
ACR d	IDC	Luminal B	TAC	CR	PR	PR	pPR
ACR d	IDC	Luminal A	AC	PD	CR	PD	pPD
ACR a	IDC	Luminal B	AC-T	PR	PR	PR	pPR
ACR b	IDC	Triple-negative	AC-T	PR	CR	PR	pCR
ACR d	IDC	Triple-negative	AC	SD	CR	SD	pSD
ACR d	IDC	Triple-negative	AC	PR	PR	SD	pPR
ACR c	IDC	Triple-negative	AC-T	CR	CR	CR	pCR
ACR b	Mixed	Luminal B	AC-T	PR	PR	PR	pPR
ACR b	IDC	Triple-negative	AC-T	PR	PR	PR	pPR
ACR c	IDC	Luminal B	AC-T	CR	PR	PR	pPR
ACR b	IDC	Luminal B	AC-TH	CR	PR	CR	pCR
ACR b	IDC	Luminal B	FAC-T	PR	PR	PR	pPR
ACR b	IDC	Triple-negative	AC-T	CR	CR	CR	pCR
ACR d	ILC	Luminal A	AC-T	SD	SD	SD	pSD
ACR c	IDC	Luminal B	AC-T	PR	PR	PR	pPR
ACR b	IDC	Luminal A	AC-T	CR	CR	PR	pPR
ACR c	IDC	Triple-negative	AC-T	CR	CR	CR	pPR
ACR c	IDC	Luminal B	TAC	CR	CR	CR	pCR
ACR a	IDC	Triple-negative	AC-T	SD	SD	SD	pSD
ACR c	IDC	Luminal A	AC-T	PR	CR	PR	pPR
ACR c	IDC	Luminal B	AC-T	CR	PR	CR	pCR
ACR d	IDC	Luminal B	AC-T	PD	PD	PD	pPD
ACR c	IDC	Luminal B	AC-T	CR	CR	CR	pCR
ACR c	Mixed	Luminal B	AC-T	CR	PR	PR	pPR
ACR b	IDC	Triple-negative	AC-T	PR	PR	PR	pPR
ACR d	IDC	Luminal A	FAC-T	PR	PR	PR	pPR
ACR c	IDC	Triple-negative	FAC-T	PR	PR	PR	pPR

NAC: neoadjuvant chemotherapy; FFDM: full-field digital mammography; CEM: contrast-enhanced mammography; MRI: magnetic resonance imaging; CR: complete response; PR: partial response; SD: stable disease; PD progressive disease.

**Table 2:** Comparison of imaging modalities and histopathological tumor.

	**Concordance (95**%** CI)**^∗^	**Correlation (p-value)** ^#^	**Mean Difference (SD)**	**LOA**
**FFDM**	0.3 (0.01-0.6)	0.3 (0.06)	0.7 (1.9)	-4.1 - 5.7
**CEM**	0.7 (0.6-0.8)	0.8 (< 0.001)	0.8 (2.5)	-2.9 - 4.5
**MRI**	0.4 (0.1-0.7)	0.5 (0.01)	1.8 (2.9)	-3.8 - 7.5

^*∗*^Lin's concordance coefficient; ^#^Pearson correlation coefficient. LOA: limits of agreement; FFDM: full-field digital mammography; CEM: contrast-enhanced mammography; MRI: magnetic resonance imaging.

**Table 3:** Performance of imaging modalities for detecting residual tumor after NAC.

	**Sensitivity**	**Specificity**	**PPV**	**NPV**	**AUC**
**FFDM**	76%	62.5%	86.4%	45.4%	0.8
**CEM**	76%	87.5%	95%	53.8%	0.8
**MRI**	92%	75%	92%	75%	0.9

NAC: neoadjuvant chemotherapy; PPV: positive predictive value; NPV: negative predictive value; AUC: area under the receiver operator characteristic curve; FFDM: full-field digital mammography; CEM: contrast-enhanced mammography; MRI: magnetic resonance imaging.

## Data Availability

The data used to support the findings of this study are available from the corresponding author upon request.

## Abbreviations

AUC: Area under the receiver operator characteristic curve
CC: Craniocaudal
CEM: Contrast-enhanced mammography
FFDM: Full-field digital mammography
IDC: Invasive ductal carcinoma
LOA: Limits of agreement
MLO: Mediolateral oblique
MRI: Magnetic resonance imaging
NAC: Neoadjuvant chemotherapy
NPV: Negative predictive value
pCR: Pathologic complete response
PPV: Positive predictive value
RD: Residual disease
US: Ultrasound.
